# A Review on the Recent Advancements of Polymer-Modified Mesoporous Silica Nanoparticles for Drug Delivery Under Stimuli-Trigger

**DOI:** 10.3390/polym17121640

**Published:** 2025-06-13

**Authors:** Madhappan Santhamoorthy, Perumal Asaithambi, Vanaraj Ramkumar, Natarajan Elangovan, Ilaiyaraja Perumal, Seong Cheol Kim

**Affiliations:** 1 School of Chemical Engineering, Yeungnam University, Gyeongsan 38541, Republic of Korea; santham83@yu.ac.kr; 2 Department of Water Supply and Environmental Engineering, Faculty of Civil and Environmental Engineering, Jimma Institute of Technology, Jimma University, Jimma P.O. Box 378, Ethiopia; drasaithambi2014@gmail.com; 3 Centre for Global Health Research, Saveetha Institute of Medical and Technical Sciences, Saveetha Medical College, Chennai 602105, India; drelangovanchem@gmail.com; 4 Department of Chemistry, School of Advanced Sciences, Vellore Institute of Technology, Chennai 600127, India; ilaiyaraja.perumal@vit.ac.in

**Keywords:** mesoporous silica, polymer modification, drug delivery carriers, stimuli-triggers, nanocarrier system

## Abstract

Mesoporous silica nanoparticles (MSNs) are gaining popularity in nanomedicine due to their large surface area, variable pore size, great biocompatibility, and chemical adaptability. In recent years, the combination of smart polymeric materials with MSNs has transformed the area of regulated drug administration, particularly under stimuli-responsive settings. Polymer-modified MSNs provide increased stability, longer circulation times, and, most crucially, the capacity to respond to diverse internal (pH, redox potential, enzymes, and temperature) and external (light, magnetic field, and ultrasonic) stimuli. These systems allow for the site-specific, on-demand release of therapeutic molecules, increasing treatment effectiveness while decreasing off-target effects. This review presents a comprehensive analysis of recent advancements in the development and application of polymer-functionalized MSNs for stimuli-triggered drug delivery. Key polymeric modifications, including thermoresponsive, pH-sensitive, redox-responsive, and enzyme-degradable systems, are discussed in terms of their design strategies and therapeutic outcomes. The synergistic use of dual or multiple stimuli-responsive polymers is also highlighted as a promising avenue to enhance precision and control in complex biological environments. Moreover, the integration of targeting ligands and stealth polymers such as PEG further enables selective tumor targeting and immune evasion, broadening the potential clinical applications of these nanocarriers. Recent progress in stimuli-triggered MSNs for combination therapies such as chemo-photothermal and chemo-photodynamic therapy is also covered, emphasizing how polymer modifications enhance responsiveness and therapeutic synergy. Finally, the review discusses current challenges, including scalability, biosafety, and regulatory considerations, and provides perspectives on future directions to bridge the gap between laboratory research and clinical translation.

## 1. Introduction

The science of administering drugs has seen amazing advances in recent decades, notably with the advent of nanotechnology. Mesoporous silica nanoparticles (MSNs) have received a lot of interest for their unusual structural features, which include a high surface area, variable pore size, and great biocompatibility [1]. MSNs are an attractive platform for drug delivery systems because they allow a variety of therapeutic substances, such as small compounds, proteins, and nucleic acids, to be loaded into their pores [2]. However, while MSNs provide effective drug encapsulation, their clinical application has been hindered by challenges related to premature drug release, low bioavailability, and lack of control over targeting specific sites in the body [3].

To address these limitations, the functionalization of MSNs with polymers has emerged as a promising strategy. Polymers, particularly those that are responsive to external or internal stimuli, can significantly enhance the performance of MSNs by imparting controlled release, increased stability, and enhanced targeting capabilities [4]. PM-MSNs combine the benefits of both materials, including MSNs’ variable porous structure and polymers’ stimuli-responsive characteristics. These hybrid systems may respond to a variety of biological stimuli, including pH, temperature, redox potential, enzymes, and external signals, such as light or magnetic fields [5]. Such technologies allow for the on-demand release of encapsulated pharmaceuticals, resulting in a more targeted therapeutic impact with fewer adverse effects.

Stimuli-responsive drug delivery systems (DDS) are particularly appealing in the context of cancer therapy, where traditional treatment strategies often lead to significant off-target effects. By utilizing polymer-modified MSNs, drugs can be released selectively in response to the unique microenvironment of tumor sites, such as acidic pH or elevated levels of specific enzymes [6]. Furthermore, the combination of multiple stimuli-responsive elements in polymeric formulations has expanded the potential for complex therapies that synergize different modes of action, such as chemotherapy combined with photothermal or photodynamic therapy [7].

This review provides an in-depth summary of current advances in PM-MSNs for drug delivery under stimulus-triggered settings. Further, the various types of polymers utilized in combination with MSNs, their methods of action, the therapeutic outcomes obtained in preclinical and clinical trials, the hurdles of moving these systems from the lab to the clinic, and future approaches for increasing the effectiveness, safety, and scalability of PM-MSN-based drug delivery are also discussed.

## 2. Mesoporous Silica Nanoparticles (MSNs)

### 2.1. Synthesis and Structural Characteristics

MSNs are a type of nanostructured material distinguished by their highly organized pore architecture, high surface area, and variable pore and particle size. These characteristics make MSNs ideal for biological applications, notably as drug delivery vehicles [8]. MSNs are generally synthesized via a sol-gel technique in the presence of structure-directing chemicals, most notably surfactants like cetyltrimethylammonium bromide (CTAB), which create micelles that guide the creation of mesopores [9].

The most widely used synthesis method is the Stöber method and its modified variants, which allow for precise control over particle size and morphology. In a typical process, tetraethyl orthosilicate (TEOS) serves as the silica precursor, which undergoes hydrolysis and condensation in an aqueous or alcoholic medium under basic or acidic conditions [10]. The choice of reaction parameters, including pH, temperature, surfactant concentration, and the presence of co-solvents or swelling agents, plays a critical role in determining the mesostructure and particle uniformity. After synthesis, the removal of the surfactant template, usually by calcination or solvent extraction, yields the final mesoporous structure. The yield of mesoporous silica synthesized by the sol-gel method using CTAB as a template typically ranges from 70% to 90%, depending on synthesis conditions, such as pH, temperature, TEOS concentration, and the method of template removal [11].

Structurally, MSNs possess a well-ordered porous network with pore diameters typically ranging from 2 to 10 nm, which can be adjusted to accommodate different therapeutic molecules. The high surface area (up to 1000 m^2^/g) and pore volume enable high drug-loading capacity [12]. MSNs can be engineered into various morphologies, including spheres, rods, cubes, and hollow structures, influencing their cellular uptake and biodistribution. Furthermore, the abundant silanol groups on the surface provide chemical handles for post-synthetic functionalization with polymers, targeting ligands, or responsive moieties [13]. This stimulus-responsiveness improves therapeutic precision by assuring drug release predominantly at the infected site, thereby reducing systemic toxicity and adverse effects. Furthermore, MSNs enable multifunctional applications, such as simultaneous administration of therapeutics and diagnostic imaging (theranostics) by co-loading imaging agents into mesopores [14]. This multifunctionality allows for real-time monitoring of drug distribution and therapy efficacy.

### 2.2. Challenges of MSN in Biological Applications

However, the direct application of MSNs in biological systems poses several critical challenges that limit clinical translation. One major concern is the colloidal instability of bare MSNs in physiological environments. Without surface modification, MSNs tend to aggregate due to high surface energy, leading to poor dispersion, rapid clearance, and uneven biodistribution. Additionally, off-target accumulation in non-diseased tissues can occur, especially in organs like the liver and spleen, owing to nonspecific protein adsorption (opsonization) and recognition by the mononuclear phagocyte system. This not only reduces therapeutic efficacy but also contributes to systemic toxicity and potential long-term adverse effects, as the biodegradability and clearance of unmodified MSNs remain insufficiently controlled. To address these issues, polymer functionalization plays a crucial role in enhancing the biological performance of MSNs. Coating MSNs with hydrophilic, biocompatible polymers such as PEG, chitosan, or zwitterionic polymers can significantly improve colloidal stability by introducing steric hindrance and preventing aggregation. Surface polymers also shield the particles from premature opsonization and immune recognition, thereby prolonging circulation time and enhancing passive tumor targeting via the enhanced permeability and retention (EPR) effect.

## 3. Polymer Modification of MSNs

### 3.1. Purpose of Polymer Functionalization

The functionalization of MSNs with polymers is crucial for improving their efficacy as advanced drug delivery systems, especially in stimuli-responsive applications. While MSNs have a large surface area, variable porosity, and superior drug-loading capacity, their bare surfaces lack the dynamic, responsive behavior required for precise control over drug release in complicated biological settings [15]. Polymer functionalization addresses this limitation by imparting stimuli-responsive capabilities, improving biocompatibility, prolonging circulation time, and enabling active targeting [16]. One of the primary purposes of polymer modification is to regulate drug release through the incorporation of “gatekeeper” systems. These polymeric gatekeepers can respond to particular physiological or environmental stimuli, including pH shifts, redox potential, temperature changes, enzyme activity, or light irradiation, allowing therapeutic drugs to be released on-demand and at precise sites. For example, pH-sensitive polymers are stable at physiological pH but undergo structural changes or breakdown in acidic tumor microenvironments, resulting in the release of encapsulated pharmaceuticals [17].

Furthermore, polymer coatings improve the biocompatibility and colloidal stability of MSNs. Hydrophilic polymers, such as polyethylene glycol (PEG), generate a hydration layer surrounding nanoparticles, limiting serum protein opsonization and inhibiting MPS clearance [18]. This “stealth” characteristic prolongs systemic circulation and enhances the possibility of tumor formation due to the increased permeability and retention (EPR) impact [16]. Functional polymers can also be coupled with targeting ligands like folic acid, peptides, or antibodies, allowing for active targeting of cancer cells that express particular receptors, enhancing treatment selectivity and decreasing off-target effects [19].

Another critical purpose of polymer functionalization is the enhancement of structural integrity and drug retention during circulation. Polymers can physically seal the mesopores or construct crosslinked networks to prevent premature drug leakage, ensuring that the therapeutic payload is protected until the proper stimulus is encountered [20]. Furthermore, PM-MSNs provide modular and multifunctional design options, allowing for the co-delivery of several therapeutic drugs or the combination of therapeutic and diagnostic (theranostic) functionalities on a single nanoplatform. This is especially beneficial in combination therapy, where polymers may be produced to respond to various inputs, resulting in sequential or synergistic drug release [21]. The polymer functionalization of MSNs is not merely a surface modification but a strategic enhancement that transforms passive carriers into intelligent, responsive drug delivery systems. By incorporating polymers with tailored responsiveness, targeting abilities, and protective functions, it is possible to create versatile nanocarriers capable of addressing the complex demands of modern precision medicine.

### 3.2. Types of Polymers Used for the Modification of MSNs

A variety of natural and synthetic polymers have been used to alter MSNs, with each chosen for their physicochemical qualities, biocompatibility, and reactivity to certain stimuli. The polymer used is critical in providing MSNs with stimuli-triggered drug release characteristics, better systemic circulation, targeted administration, and increased stability [22]. Among the most extensively used polymers is PEG, a hydrophilic, biocompatible polymer widely recognized for its “stealth” properties. PEGylation of MSNs enhances their colloidal stability, reduces protein adsorption, and prolongs blood circulation time by evading the mononuclear phagocyte system [23]. Though PEG is not inherently stimuli-responsive, it is often used in combination with other responsive polymers or cleavable linkers to achieve triggered release.

pH-responsive polymers such as PAA, poly(L-histidine), and chitosan are commonly utilized to exploit the acidic microenvironment of tumors or endosomes [24]. These polymers undergo protonation-induced swelling or solubilization under acidic conditions, leading to the opening of mesopores and the release of cargo. Natural polymers like chitosan offer additional benefits, including mucoadhesiveness, biodegradability, and antimicrobial activity, making them attractive for mucosal or localized delivery [25].

Thermoresponsive polymers, such as PNIPAM, undergo phase transitions in response to temperature variations. PNIPAM undergoes a reversible sol-gel transition at its lower critical solution temperature (LCST), enabling temperature-controlled drug release [26]. When conjugated onto MSNs, PNIPAM can act as a thermally responsive gatekeeper that collapses or expands in response to temperature fluctuations, which is particularly useful in hyperthermia-assisted cancer therapy [27].

Redox-responsive polymers, especially those with a disulfide bridge, such as poly(disulfide-ethylene glycol) or thiol-functionalized PEG, are disrupted in the presence of elevated intracellular GSH levels, which are frequent in cancer cells [28]. These polymers provide selective intracellular release while remaining stable in extracellular conditions, improving the therapeutic index. Enzyme-sensitive polymers, such as gelatin, dextran, and PCL derivatives, respond to overexpressed enzymes like matrix metalloproteinases (MMPs) or esterases in diseased tissues [29]. These polymers degrade in the presence of target enzymes, enabling enzyme-specific release mechanisms. In addition, light-responsive polymers incorporating photo-cleavable or photo-isomerizable moieties (e.g., azobenzene or coumarin) allow for non-invasive, externally controlled drug release under UV or NIR light [30]. Such systems are particularly promising for spatiotemporally precise therapies.

### 3.3. Functionalization Strategies for the Modifications of MSNs

Functionalization methods are critical in the effective modification of MSNs with polymers to create smart, stimuli-responsive drug delivery systems. These techniques not only affect the physicochemical interaction between the polymer and the MSN surface, but they also influence drug encapsulation efficiency, stimulus responsiveness, and biological performance [31]. Broadly, polymer functionalization on MSNs can be achieved through covalent grafting, electrostatic adsorption, physical entrapment, and “grafting-from” or “grafting-to” techniques, each offering distinct advantages depending on the application (Table 1).

One of the most widely used approaches is covalent grafting, where polymers are chemically bonded to the MSN surface via silanol groups [32]. This method ensures strong and stable attachment, minimizing the risk of polymer detachment during circulation. Covalent linkage can be established using silane coupling agents like (3-aminopropyl)triethoxysilane (APTES) or (3-mercaptopropyl)trimethoxysilane (MPTMS), which introduce reactive amine or thiol groups on the MSN surface, subsequently reacting with functional groups on the polymer chains [33]. This strategy is particularly beneficial for stimuli-responsive systems, such as redox- or pH-sensitive polymers, where structural integrity is crucial for controlled drug release.

In contrast, electrostatic adsorption is based on non-covalent interactions between oppositely charged polymers and MSNs. For example, positively charged chitosan can be electrostatically adsorbed to the negatively charged silica surface [34]. This method is simpler and does not require complex chemistry, but may offer less stability under physiological conditions. However, it is suitable for transient modifications or systems where rapid response and easy degradation are desired [35].

The “grafting-from” technique includes in situ polymerization of monomers that begin on the MSN surface. This technology allows for the creation of dense and homogenous polymer brushes with adjustable thickness and composition [36]. Surface-initiated atom transfers radical polymerization (SI-ATRP) and reversible addition-fragmentation chain transfer (RAFT) polymerization are popular methods for this purpose. The “grafting-from” method is advantageous for tailoring stimuli-responsive behavior and designing multifunctional surfaces [37].

Alternatively, the “grafting-to” strategy involves attaching pre-formed polymer chains to the MSN surface via reactive end groups. Although steric hindrance may limit grafting density, this method allows for the use of well-characterized polymers with predefined molecular weights and functionalities [38]. In addition, physical entrapment or encapsulation of polymers into or around the MSN structure can be used to form core-shell nanostructures. These shells can act as gatekeepers or protective barriers, especially when employing thermoresponsive or enzymatically degradable polymers [39].

Green synthesis and treatment methods for polymer modification emphasize the use of sustainable, non-toxic, and energy-efficient techniques that align with the principles of green chemistry. These approaches replace conventional chemical processes, which often involve hazardous reagents and generate toxic waste, with eco-friendly alternatives [40]. One common method is the use of plant extracts, bacteria, or fungi as natural reducing and capping agents in the synthesis of nanomaterials for polymer modification. These biological agents are rich in polyphenols, flavonoids, and enzymes that facilitate in situ reactions under mild conditions. Solvent-free techniques or the use of green solvents such as water, ethanol, or supercritical CO_2_ further enhance the environmental compatibility of the process. Additionally, microwave and ultrasound-assisted synthesis reduce reaction time and energy consumption while improving yield and material properties [41].

**Table 1:** The advantages and limitations of different polymer functionalization methods.

Method	Advantages	Limitations	Refs.
Covalent Grafting	- Strong, stable attachment - Good control over surface chemistry - Long circulation stability	- May block pores, reducing drug loading - Requires multi-step reactions and harsh conditions - Difficult to reverse	[32]
Electrostatic Adsorption	- Simple and mild process - Reversible binding - Suitable for sensitive biomolecules	- Weak interaction, prone to desorption - Sensitive to pH and ionic strength - Lower long-term stability	[34]
Physical Entrapment	- Easy to perform - No chemical modification required - Minimal reaction steps	- Poor control over release and coating - Risk of premature polymer leaching - Weak interaction with the MSN surface	[31]
Grafting-To	- Pre-synthesized polymers with defined properties - Good for functional polymer integration	- Low grafting density due to steric hindrance - Limited surface coverage and uniformity	[38]
Grafting-From	- High grafting density - Precise control over chain length and density - Uniform coating	- Complex synthesis - Requires initiators and controlled polymerization - Potential toxicity from residual catalysts	[36]

### 3.4. Characterization of Polymer Functionalized MSNs

Comprehensive characterization of PM-MSNs is critical for confirming successful surface modification, evaluating structural integrity, and assessing their suitability for stimuli-responsive drug delivery applications. Various physicochemical and morphological characterization techniques are employed to validate polymer grafting, determine particle stability, and understand interactions between the polymer and the MSN matrix [42].

X-ray diffraction (XRD) is a vital technique for assessing the structural order of MSNs before and after polymer functionalization. Typically, MSNs exhibit well-defined low-angle reflections (e.g., (100), (110), and (200) planes) associated with their hexagonal mesostructure. After polymer grafting or coating, a slight reduction in peak intensity or partial peak broadening may occur, indicating partial pore filling or surface coverage without significant disruption of the mesostructure. The absence of new crystalline peaks confirms the amorphous nature of the polymer [43].

**Figure 1:**
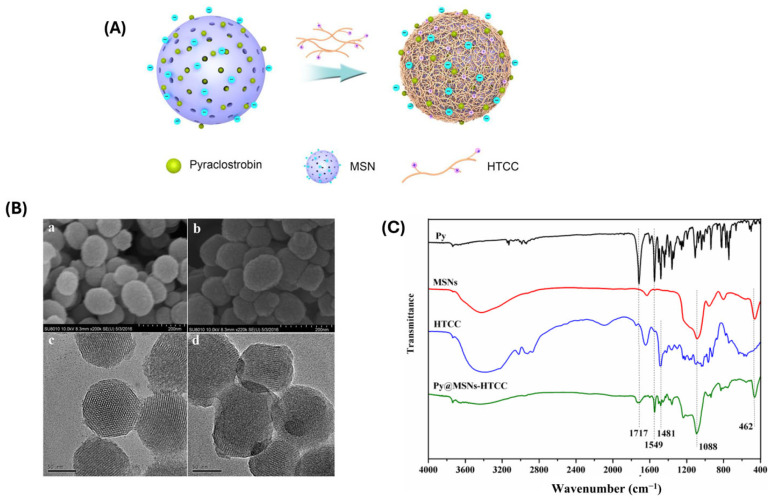
(**A**) Preparation of pyraclostrobin-loaded HTCC-capped mesoporous silica nanoparticles (MSNs); (**B**) (SEM) images of MSNs (**a**), pyraclostrobin-loaded HTCC-capped MSNs (**b**), Transmission electron microscopy (TEM) images of MSNs (**c**), and pyraclostrobin-loaded HTCC-capped MSNs (**d**); (**C**) FTIR spectra of pyraclostrobin (Py), MSNs, HTCC and Py@MSNs-HTCC. ref. [44].

Transmission electron microscopy (TEM) and scanning electron microscopy (SEM) are routinely used to analyze particle morphology and confirm the preservation of mesoporous structures after polymer coating. TEM can reveal core-shell architecture and uniformity of polymer layers, while SEM provides insight into surface topology and size distribution. Dynamic light scattering (DLS) and zeta potential measurements are essential for assessing the hydrodynamic diameter and surface charge, respectively [44] (Figure 1A–C). Polymer functionalization often leads to an increase in particle size and alters surface charge, which serves as indirect evidence of successful conjugation. Zeta potential changes also indicate surface charge reversibility under specific stimuli, which is relevant for pH- or redox-responsive systems [45]. The particle size range of PM-MSNs typically falls between 80 to 200 nanometers (nm). The core MSN structure generally ranges from 50 to 150 nm, while polymer modification, using materials like polyethylene glycol (PEG), chitosan, or thermoresponsive polymers, adds an outer layer that increases the overall diameter. The final size depends on the type, thickness, and molecular weight of the polymer used.

**Figure 2:**
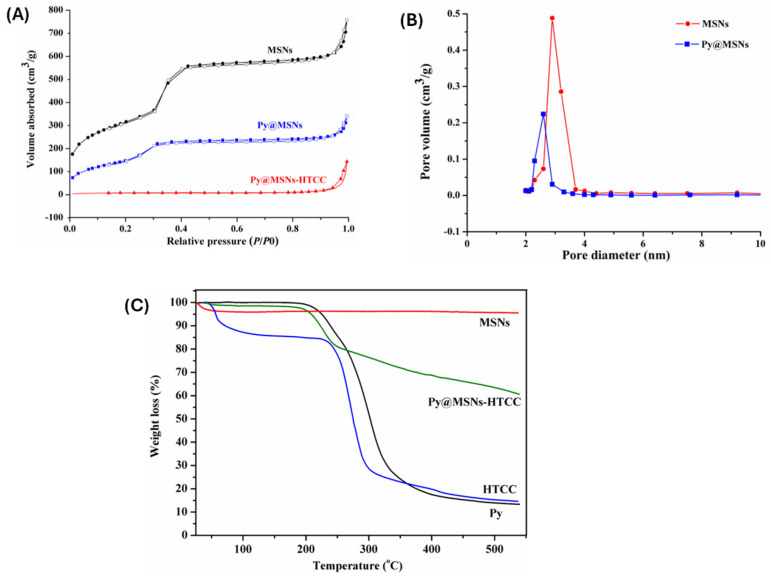
(**A**,**B**) Nitrogen adsorption–desorption isotherms of MSNs, Py@MSNs and Py@MSNs-HTCC, and pore-size-distribution curves of MSNs and Py@MSNs; (**C**) TGA of pyraclostrobin, MSNs, HTCC and Py@MSNs-HTCC. ref. [44].

Fourier-transform infrared spectroscopy (FTIR) and Raman spectroscopy help identify characteristic functional groups for both the polymer and silica framework. The appearance of new absorption bands corresponding to polymeric moieties (e.g., –COOH, –NH_2_, –SH, or –PEG) confirms chemical bonding or adsorption onto MSN surfaces. Nitrogen adsorption–desorption isotherms analyzed via the BET (Brunauer–Emmett–Teller) method assess textural properties, such as surface area, pore volume, and pore diameter. After polymer grafting or encapsulation, a noticeable decrease in surface area and pore volume typically indicates pore coverage or blockage, validating successful modification. In addition, thermogravimetric analysis (TGA) quantifies the polymer content by measuring weight loss associated with the organic component, offering insight into functionalization density [46] (Figure 2A–C) (Table 2). X-ray photoelectron spectroscopy (XPS) further confirms the chemical environment and bonding interactions at the surface level [47]. For stimuli-responsive systems, UV-vis spectroscopy, and fluorescence spectroscopy, differential scanning calorimetry (DSC) is employed to evaluate polymer responsiveness (e.g., thermal transitions or light-triggered changes) [48]. These instrumental characterization techniques provide an overall understanding of polymer–MSN hybrids, ensuring that the modifications are stable, reproducible, and functionally responsive to the intended stimuli.

Surface-modified MSNs demonstrate excellent compatibility with biological systems due to their tailored surface properties. Coating MSNs with biocompatible polymers such as PEG, chitosan, or dextran reduces cytotoxicity, enhances aqueous stability, and prevents non-specific protein adsorption. These modifications also improve circulation time and cellular uptake while minimizing immune recognition [49].

For FDA approval and commercialization of MSNs, a series of standardized cytotoxicity tests are required to ensure safety. Common in vitro tests include MTT, LDH release, trypan blue exclusion, and live/dead staining assays, which evaluate cell viability, membrane integrity, and metabolic activity. These are followed by in vivo toxicity studies in animal models to assess biodistribution, organ accumulation, and immunogenicity. Upon successful preclinical testing, MSNs must undergo clinical trials: Phase I evaluates safety in humans, Phase II assesses therapeutic efficacy and optimal dosing, and Phase III confirms effectiveness in larger populations.

## 4. Advantages of Drug Delivery

MSNs have several distinguishing characteristics that make them ideal for drug delivery applications. One of the most appealing aspects of MSNs is their huge surface area and pore volume, which enable the effective loading of a wide range of therapeutic agents, including hydrophobic drugs, proteins, peptides, and nucleic acids [50,51]. The advantage of MSNs lies in their excellent biocompatibility and low toxicity. Silica is a biologically safe material, and the degradation product, orthosilicic acid, is naturally excreted from the body [52]. Numerous in vitro and in vivo investigations have shown that MSNs have low toxicity at therapeutic doses, making them intriguing candidates for clinical translation [53].

Importantly, MSNs may be programmed to release their cargo in response to particular internal or external stimuli, including pH, redox conditions, enzymes, temperature, light, or magnetic fields [54]. This stimulus-responsiveness improves therapeutic precision by assuring drug release predominantly at the infected site, reducing systemic toxicity and adverse effects.

### Classification of Stimuli

Stimuli-responsive drug release mechanisms are critical in the development of polymer-modified MSNs for intelligent and controlled drug delivery. These systems are designed to release therapeutic cargo in response to specific physiological or externally applied triggers, increasing treatment efficacy while reducing systemic toxicity [55,56]. Functional polymers are typically grafted onto or around MSNs to act as gatekeepers, capping the mesopores and preventing premature drug leakage. Upon exposure to a particular stimulus, these polymers undergo structural or chemical changes such as swelling, degradation, or cleavage, allowing the drug to be released precisely at the target site [57].

**Figure 3:**
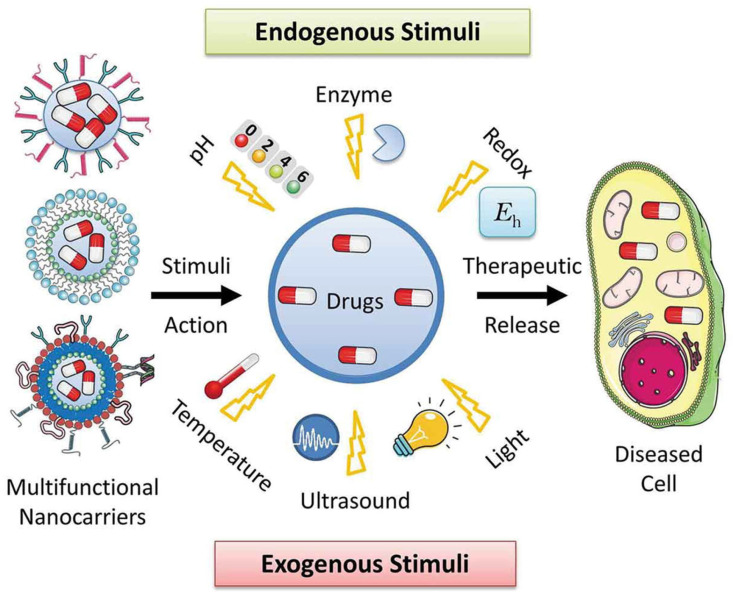
Schematic representation of different stimuli-responsive multifunctional nanocarriers (polymeric nanoparticles, liposomes, nanostructured lipid carriers, etc.) for targeted drug delivery applications. Reproduced with permission from Taylor & Francis, 2021, ref. [61].

Endogenous stimuli, including pH, redox potential, and enzymatic activity, are commonly exploited in cancer therapy due to their abnormal levels in tumor microenvironments. For example, pH-responsive polymers like PAA or chitosan dissolve or swell in acidic conditions (pH ~5–6), triggering drug release inside tumor tissues or endosomes [58]. Redox-responsive systems utilize disulfide-containing polymers that degrade in the presence of elevated intracellular glutathione (GSH), ensuring release specifically within cancer cells. Enzyme-sensitive polymers, such as gelatin or dextran, are degraded by overexpressed enzymes like matrix metalloproteinases (MMPs), facilitating site-specific delivery. Exogenous stimuli provide external control over drug release and include temperature, light, and magnetic fields. Thermoresponsive polymers, such as PNIPAM, have reversible phase transitions around body temperature, making them excellent for hyperthermia-induced release [59]. Light-responsive systems incorporate photo-cleavable linkers or photo-isomerizable units (e.g., azobenzene), allowing precise spatiotemporal control over drug release upon exposure to UV or near-infrared (NIR) light [60]. Magnetic field-responsive systems frequently use magnetic nanoparticles that create localized heat when exposed to an alternating magnetic field, causing the release of thermoresponsive polymer shells. The integration of stimuli-responsive polymers with MSNs provides a highly tunable platform for on-demand, site-specific drug delivery [61] (Figure 3). This smart release behavior not only improves therapeutic outcomes by maximizing drug concentration at the disease site but also significantly reduces off-target effects, offering great promise for personalized and precision medicine.

Stimuli-responsive drug release methods in PM-MSNs are intended to produce regulated and targeted therapeutic drug delivery in response to certain internal or external stimuli [41]. These methods use functional polymers as gatekeepers, which block the MSN pores and undergo structural or chemical modifications in response to stimuli, allowing drug release [62]. These stimuli-responsive processes improve drug release selectivity, minimize systemic adverse effects, and increase therapeutic efficacy, making PM-MSNs ideal for smart drug delivery systems.

Stimulus-responsive drug delivery from PM-MSNs involves both chemical and physical mechanisms that enable controlled and targeted drug release. MSNs possess a high surface area and tunable pore sizes, allowing efficient drug loading within their mesopores. These pores are capped or coated with stimuli-responsive polymers that act as gatekeepers. At the chemical level, these polymers respond to internal (e.g., pH, redox, enzymes) or external (e.g., temperature, light) stimuli by undergoing specific reactions or conformational changes. For example, in acidic tumor environments, polymers with ionizable groups (like –COOH or –NH_2_) undergo protonation or deprotonation, disrupting electrostatic interactions and triggering polymer swelling or dissolution [63]. Redox-responsive systems use disulfide linkers that cleave in the presence of elevated intracellular glutathione, detaching the polymer and opening the pores. At the physical level, these chemical changes result in polymer swelling, shrinking, or detachment, exposing the nanopores and facilitating drug diffusion. Thermoresponsive polymers like PNIPAM collapse above their lower critical solution temperature, unblocking the pores. Similarly, photo-responsive systems use light to cleave chemical bonds or induce isomerization, altering the polymer structure and enabling drug release. Together, these chemical transformations and physical changes ensure site-specific, stimuli-triggered, and temporally controlled drug delivery, enhancing therapeutic efficacy while minimizing systemic toxicity.

## 5. Recent Advancements in Polymer-Modified MSNs for Controlled Drug Delivery

In recent years, PM-MSNs have seen substantial progress in the creation of smart drug delivery systems that can respond to both internal and external stimuli [64]. These developments are motivated by the desire for greater therapeutic accuracy, lower systemic toxicity, and better patient outcomes. Researchers created multi-responsive MSNs functionalized with polymers that respond to a variety of internal stimuli seen in tumor microenvironments, including pH, redox potential, and enzymatic activity. For example, dual-responsive systems incorporating pH- and redox-sensitive polymers have demonstrated improved site-specific release, leveraging acidic pH and elevated glutathione levels to ensure intracellular drug release [65]. Additionally, enzyme-sensitive coatings using substrates like gelatin or hyaluronic acid have enabled selective release in response to tumor-associated enzymes.

On the external stimulus front, light- and temperature-responsive polymer coatings have gained attention for their precise control and non-invasive activation. For instance, near-infrared (NIR)-responsive PNIPAM-MSNs enable on-demand drug release upon photothermal heating [66]. Similarly, light-cleavable polymers integrated with photo-responsive linkers allow for controlled drug release using spatially targeted irradiation. Recent studies have also explored magnetically responsive MSNs, where magnetic nanoparticles are encapsulated or attached to MSNs and combined with thermosensitive polymers to facilitate drug release under alternating magnetic fields [67].

### Classification of Various Stimuli-Triggered Drug Delivery from the PM-MSNs

(i) pH-responsive systems

pH-responsive drug delivery is one of the most widely researched and implemented techniques in the creation of PM-MSNs for targeted and controlled drug release. The technique makes use of the pH differences between healthy and tumor tissues, including the slightly acidic milieu of tumors (pH ~6.5–6.8), endosomes (pH ~5.5–6), and lysosomes (pH ~4.5–5.5), as opposed to the neutral physiological pH (~7.4) of blood and normal tissues [68]. This gradient permits the selective activation of pH-sensitive polymer coatings on MSNs, resulting in site-specific drug release, lowering off-target toxicity, and improving therapeutic effectiveness.

To achieve pH responsiveness, MSNs are commonly functionalized with polymers that undergo conformational changes, solubility shifts, or cleavage of acid-labile bonds in acidic environments. Polymers such as PAA, poly(L-histidine), chitosan, and polyaniline are widely used due to their ability to protonate or swell at lower pH levels [69]. For example, PAA can swell and uncork the MSN pores when protonated under acidic conditions, enabling the release of encapsulated drugs. Similarly, chitosan, a natural polysaccharide, dissolves under acidic conditions and has been used to form pH-responsive gates on MSNs.

**Figure 4:**
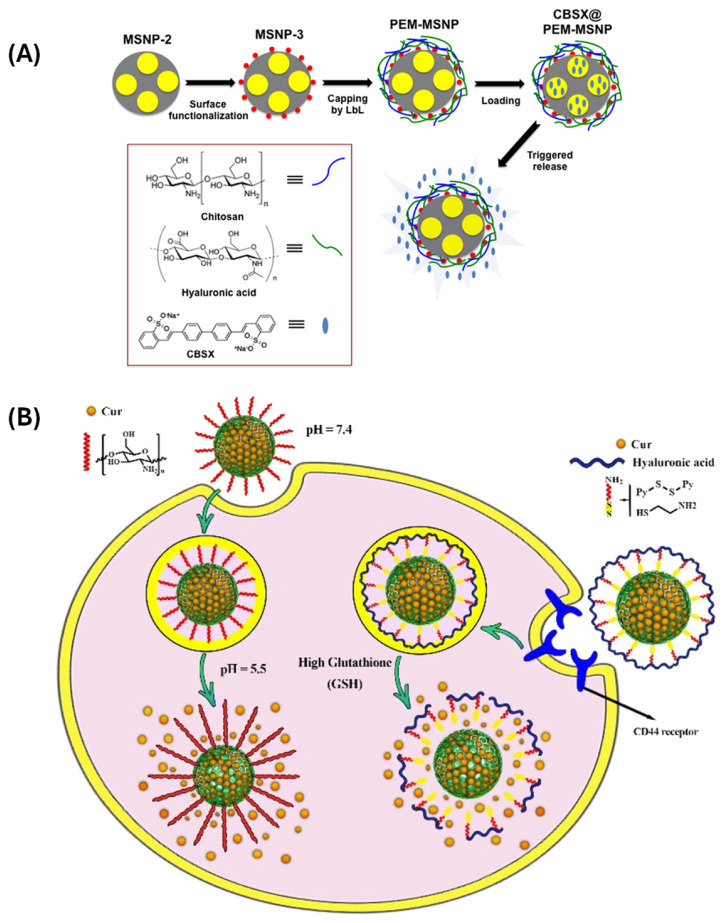
(**A**) pH-stimuli-responsive drug delivery from the CBSX-loaded mesoporous silica based nanocontainers. Reproduced with permission from Elsevier, 2016, ref. [70]. (**B**) curcumin-loaded MSNs, glutathione (GSH)-responsive curcumin-loaded MSNs, and their release mechanisms. ref. [76].

Yilmaz [70] developed polyelectrolyte multilayer coated MSNs (PEM-MSNPs) for the encapsulation of an industrially utilized OB, 4,4′-distyrylbiphenyl sulfonate sodium salt (CBSX), and investigated its pH-controlled release for optical bleaching of cellulose fibers. The controlled release of CBSX from nanocontainers in response to pH is measured with a UV-vis and fluorescence spectrophotometer in water, and virtually all the encapsulated CBSX is released within 2 h at pH 7 (Figure 4A). Advanced systems have also included acid-labile linkers such as hydrazone, acetal, or imine bonds, which deteriorate in acidic conditions, causing pharmaceuticals to be released or polymer shells to dissolve [71]. This method gives fine control over drug release kinetics and improves payload protection while in circulation. Recent studies have demonstrated the co-delivery of chemotherapeutics and pH-sensitive polymers in a single MSN platform, enabling synergistic effects in cancer therapy. Furthermore, the integration of pH-responsive behavior with other stimuli (e.g., redox or temperature) has resulted in dual or multi-responsive systems for enhanced specificity and responsiveness in complex tumor microenvironments [72].

(ii) Redox-responsive systems

Redox-responsive drug delivery systems have been developed as a potent technique for obtaining regulated and site-specific therapeutic release, especially in cancer treatment [73]. This technique takes advantage of the large redox potential difference between the external and intracellular surroundings. While the extracellular milieu, including blood plasma, maintains a relatively low concentration of reducing agents such as glutathione (GSH) (~2–10 µM), the intracellular environment, especially in cancer cells, contains substantially higher GSH levels (up to 10 mM). This redox gradient forms the basis for designing PM-MSNs that respond specifically to intracellular reducing conditions [74].

Redox-responsive MSNs are typically constructed by incorporating disulfide bonds (-S-S-) into the polymeric coatings or pore-capping structures. These disulfide linkages remain stable in the bloodstream but are rapidly cleaved in the reductive intracellular environment, leading to the detachment or degradation of the polymer layer and the consequent release of the encapsulated drug [75]. The in vitro release performance of Cur for chitosan-functionalized MSNs was investigated as the pH of the medium decreased (from 7.4 to 5.5). In acidic environments, the amino groups of chitosan on the nanocarrier’s surface are protonated, transforming it into a cationic polyelectrolyte. As the pH increased, amino group deprotonation increased, whereas polymer chain repulsion and water absorption decreased, allowing chitosan polymeric chains to shorten and form layers around MSNs. The loaded Cur cannot be released because layers of chitosan polymer chains have been coated over the porous surface of MSNs (Figure 4B) [76]. Commonly used polymers include thiolated polyethylene glycol (PEG-SS), disulfide-crosslinked poly(amidoamine) dendrimers, and other synthetic polymers containing cleavable disulfide moieties. This technique minimizes premature drug leakage during circulation and increases intracellular drug accumulation, hence enhancing therapeutic efficacy and lowering systemic toxicity. The prepared system showed almost no effect on the cell growth of LO2 and HepG2 cells, demonstrating the good biocompatibility. However, when HepG2 cells were treated with PAA-cys-TOS/MTX for 24 h, cell viability is significantly decreased to 74% (0.391 μg mL^−1^), 68% (0.781 μg mL^−1^), 53% (1.563 μg mL^−1^), 40% (3.125 μg mL^−1^), 26% (6.250 μg mL^−1^), 15% (12.500 μg mL^−1^), 10% (25.000 μg mL^−1^), and 5% (50.000 μg mL^−1^), respectively, of its initial viability due to the significant release of drug from the cellular microenvironments [77]. Furthermore, multifunctional polymer networks sensitive to reductive circumstances can be designed to enable the co-delivery of several therapeutic agents, including small-molecule drugs and nucleic acids.

Recent advancements include the development of dual-responsive systems, integrating redox sensitivity with other triggers such as pH, temperature, or enzyme activity. These hybrid systems offer a higher level of control and specificity, especially in the heterogeneous microenvironments of solid tumors [78]. Additionally, redox-responsive MSNs have shown promise in combination therapies, where redox-triggered release can synergize with photothermal or photodynamic effects for enhanced tumor suppression.

(iii) Enzyme-responsive systems

Enzyme-responsive drug delivery is an advanced strategy that leverages the aberrant expression or overactivity of specific enzymes in pathological environments, particularly in cancer, inflammation, and infection sites, to achieve targeted and controlled drug release [79]. PM-MSNs equipped with enzyme-cleavable polymers or linkers have emerged as powerful carriers for such stimuli-responsive systems. These smart nanocarriers remain stable during systemic circulation but undergo structural changes upon exposure to target enzymes, enabling site-specific and on-demand drug release. For instance, the hybrid LMSNs as nanocarriers for CoQ10 (DC-TPGS-LMSNs@CoQ10) show higher cellular uptake efficiency and the resulting ROS inhibition effect, which can be taken up by cells via caveolae-mediated route, macropinocytosis, and a unique bile acid uptake pathway mediated by ASBT. Efficient delivery of DC-TPGS-LMSNs is further illustrated by an in vivo intestinal uptake and pharmacokinetics study, in which DC-TPGS-LMSNs@CoQ10 exhibits higher absorption efficiency, longer circulation time, and more enhanced bioavailability [80].

**Figure 5:**
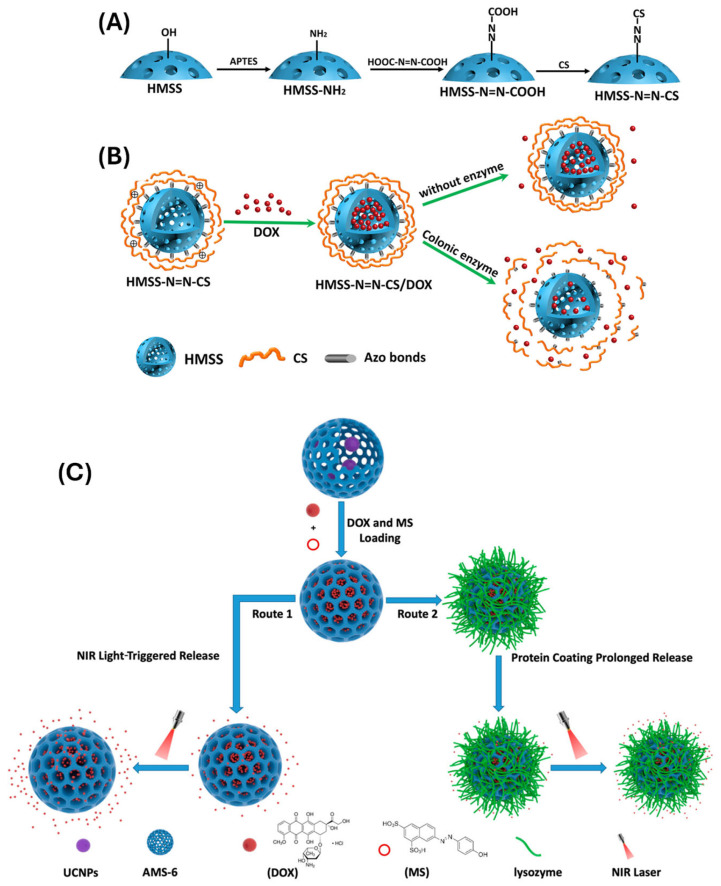
(**A**) Schematic illustration of a preparation process of HMSS–N=N–CS and (**B**) the drug loading and enzyme-responsive release of HMSS–N=N–CS/DOX in response to colon enzyme. ref. [82]. (**C**) Upconversion nanoparticles coated with mesoporous silica for controlled release under enzyme and NIR light. ref. [90].

Several enzymes have been used for this purpose, including matrix metalloproteinases (MMPs), cathepsins, esterases, and proteases, which are commonly overexpressed in tumor tissues or inflamed regions. MMP-sensitive peptides or polymers can be grafted onto the MSN surface, causing MMP cleavage to dislodge the polymer gatekeeper and release the loaded drugs. Cai et al. [81] developed a colon-specific enzyme-responsive DDS based on an HMSS material (HMSS-N=N-CS) using a selective etching method. The polymer CS was linked to the surface of HMSS by azo linkages to operate as a gatekeeper, blocking the HMSS apertures. Enzymes can break the azo linkages between HMSS and CS, causing CS to separate from the HMSS apertures. DOX served as the model drug, and in vitro drug release tests revealed enzyme-responsive release in the presence of colonic enzymes (Figure 5A,B). Similarly, esterase-responsive polymers, such as polycaprolactone (PCL) or poly(β-amino esters), degrade in response to intracellular esterases, enabling drug release within endosomes or lysosomes [82].

Natural polymers, like gelatin, hyaluronic acid, and dextran, have also been used due to their inherent biodegradability and sensitivity to enzymatic hydrolysis. These polymers can form coatings or hydrogel shells around MSNs, which are selectively degraded by enzymes, triggering cargo release [83]. For instance, hyaluronic acid not only serves as an enzyme-sensitive material but also provides active targeting to CD44 receptors overexpressed on many cancer cells, thus enhancing delivery specificity. Recent advances in this field have resulted in dual or multi-enzyme-responsive systems that respond to combinations of enzymes or other stimuli, such as pH or redox potential. These systems provide increased specificity, lowering the risk of off-target effects and enhancing treatment results [84].

The enzyme-responsive PM-MSNs provide a highly selective and effective platform for targeted drug delivery. These nanocarriers, which use the enzymatic signature of sick tissues, can provide regulated release profiles, increase bioavailability, and reduce systemic toxicity, making them intriguing tools for next-generation precision medicine [85].

(iv) Light-triggered systems

Light-responsive drug delivery systems based on PM-MSNs have emerged as a cutting-edge technique for controlling drug release in both space and time. These systems use light, particularly in the ultraviolet (UV), visible, or near-infrared (NIR) ranges, to cause chemical or physical changes in light-sensitive polymers or linkers functionalized on the surface of MSNs [86]. By directing light to target tissues, these technologies enable non-invasive, on-demand therapeutic release, reducing off-target effects and increasing the effectiveness of treatment.

Several types of photo-responsive mechanisms have been employed. A typical approach encompasses the use of photo-cleavable linkers, such as o-nitrobenzyl or coumarin derivatives, which are incorporated into polymer chains or gatekeeper structures. Upon light irradiation, these linkers break down, leading to the detachment of the polymer and opening of the MSN pores [87]. This mechanism enables precise control over when and where drug release occurs.

In recent years, there has been growing interest in using NIR-responsive systems, which offer deeper tissue penetration and lower phototoxicity compared to UV light. This is often achieved by integrating photothermal agents, such as gold nanorods, upconversion nanoparticles, or graphene oxide, with MSNs [88]. When exposed to NIR light, these agents convert light into heat, which then triggers thermoresponsive polymers (e.g., PNIPAM) to collapse and release the drug payload. Huang and colleagues [89] developed a two-way controlled drug-release system that can be accelerated and sustained by stimulating near-infrared light (NIR) and covering it with proteins. The system was built with upconversion nanoparticles (UCNPs), mesoporous silica as the core-shell structure, and protein lysozyme coating. Their findings revealed that a new DRS capable of smart regulation may increase or block drug release under NIR light and protein coating, respectively. Another strategy involves using photo-isomerizable compounds like azobenzene, which can reversibly switch between cis and trans conformations upon light exposure (Figure 5C) [90]. This molecular movement can be harnessed to control pore gating in MSNs, allowing repeatable and reversible drug release cycles. Recent advancements have also explored multi-stimuli responsive systems that combine light with pH, redox, or enzymatic triggers, enabling enhanced precision in complex biological environments.

(v) Thermo-responsive systems

Thermo-responsive drug delivery systems have received a lot of interest in recent years because of their capacity to release therapeutic drugs in response to temperature changes, giving users spatial and temporal control over drug release [91]. When incorporated with MSNs, thermo-responsive polymers can act as gatekeepers that block or unblock the nanopores depending on the surrounding temperature. This system is particularly attractive for cancer therapy, where localized hyperthermia (typically 40–45 °C) can be applied externally or induced internally (e.g., via magnetic fields or near-infrared irradiation) to trigger drug release specifically at the tumor site [92].

**Figure 6:**
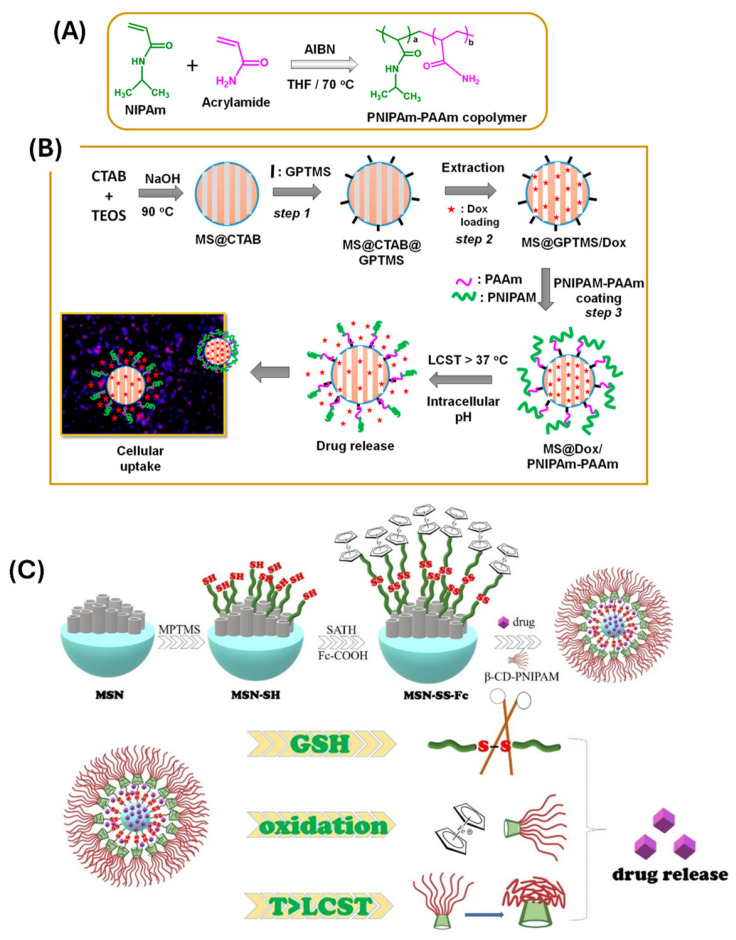
Schematic representation of the (**A**) synthesis of the PNIPAm-PAAm copolymer and (**B**) the MS@Dox/PNIPAm-PAAm NPs’ synthesis, surface modifications, drug loading, and release behavior. ref. [93]. (**C**) Formation of MSN-SS-Fc@β-CD-PNIPAM nanocarriers and a schematic illustration of drug release. ref. [94].

PNIPAM is a popular thermo-responsive polymer with a low critical solution temperature (LCST) of around 32 °C. Below this temperature, PNIPAM becomes hydrophilic and bloated, creating a barrier across the MSN pores. When the temperature exceeds its LCST, the polymer becomes hydrophobic and collapses, opening the pores and permitting drug release. Thirupathi and colleagues [93] described a thermo-responsive copolymer (PNIPAm-PAA) coated MSNs. The drug delivery tests are conducted in vitro at various pH (7.4, 6.5, and 5.0) and temperatures (25 °C and 42 °C, respectively). The surface conjugated copolymer (PNIPAm-PAAm) operates as a gatekeeper below the lower critical solution temperature (LCST) (<32 °C) and as a collapsed globule structure above LCST (>32 °C), allowing for regulated drug administration from the MS@PNIPAm-PAAm system (Figure 6A). Moreover, MS@PNIPAm-PAAm NPs were tested at the sample doses of 0, 25, 50, 100, 150, and 200 µg/mL, without or with Dox loading, using MDA-MB-231 cells. The cell viability of MS@PNIPAm-PAAm NPs without Dox loading had ~90% cell viability in all the tested concentrations. MS@Dox/PNIPAm-PAAm NPs were treated for 5 h with MDA-MB-231 cells at a sample dose of 10 μg/mL, showing the cellular uptake and consequent internalization of the MS@Dox/PNIPAm-PAAm NPs into the MDA-MB-231 cells. The LCST can be finely tuned by copolymerizing PNIPAM with other monomers, enabling customization for specific therapeutic applications.

Recent studies have demonstrated hybrid systems that combine PNIPAM with other stimuli-responsive elements (e.g., pH, redox, or light), resulting in multi-responsive nanocarriers with improved specificity and performance. For instance, dual thermo- and redox-responsive systems can offer highly selective intracellular drug release in tumor cells, which exhibit both elevated temperatures (via external induction) and high glutathione levels. Guo et al. [94] created a temperature, glutathione (GSH), and H_2_O_2_ sensitive composite nanocarrier (MSN-SS-Fc@β-CD-PNIPAM) using β-cyclodextrin-poly(N-isopropylacrylamide) (β-CD-PNIPAM) star polymer capped ferrocene modified mesoporous silica nanoparticles (MSN-SS-Fc). The mesoporous silica was modified by ferrocene (Fc) via a disulfide bond (-SS-) to generate an oxidizing and reducing site. Doxorubicin (DOX) and Naproxen (NAP) were loaded into nanocarriers as model drugs to study drug loading and release behavior. The release of medicines from nanocarriers was improved by increasing the GSH, H_2_O_2_ concentration, or solution temperature (Figure 6B). Moreover, MSNs have been functionalized with thermo-sensitive hydrogels or block copolymers that undergo sol-gel transitions upon heating, providing another mechanism for controlled drug release [95]. These materials can form a protective matrix that stabilizes the nanocarrier in circulation and rapidly releases the drug upon localized heating. Thus, the thermo-responsive PM-MSNs offer a promising strategy for externally controlled, on-demand drug delivery. Their tunable response, non-invasiveness, and compatibility with other trigger mechanisms make them highly adaptable for advanced therapeutic applications, especially in precision oncology [96].

(vi) Ultrasound-responsive systems

Ultrasound-responsive polymer-coated MSNs are a potential platform for non-invasive, spatiotemporally controlled drug delivery. Ultrasound has various benefits as an external stimulus, including deep tissue penetration, minimum invasiveness, and the capacity to modify biological barriers such as cell membranes and vasculature [97]. When paired with MSNs, which have a large surface area, variable pore size, and superior drug-loading capacity, ultrasound-responsive devices can considerably improve therapeutic efficacy while minimizing systemic toxicity. The integration of ultrasound-sensitive polymers, such as thermosensitive or cavitation-responsive compounds, onto the surface of MSNs enables fine control over drug release kinetics [98]. Following ultrasonic exposure, these polymers undergo structural changes such as phase transition, polymer disintegration, or pore uncapping, resulting in the rapid and localized release of encapsulated drugs.

**Figure 7:**
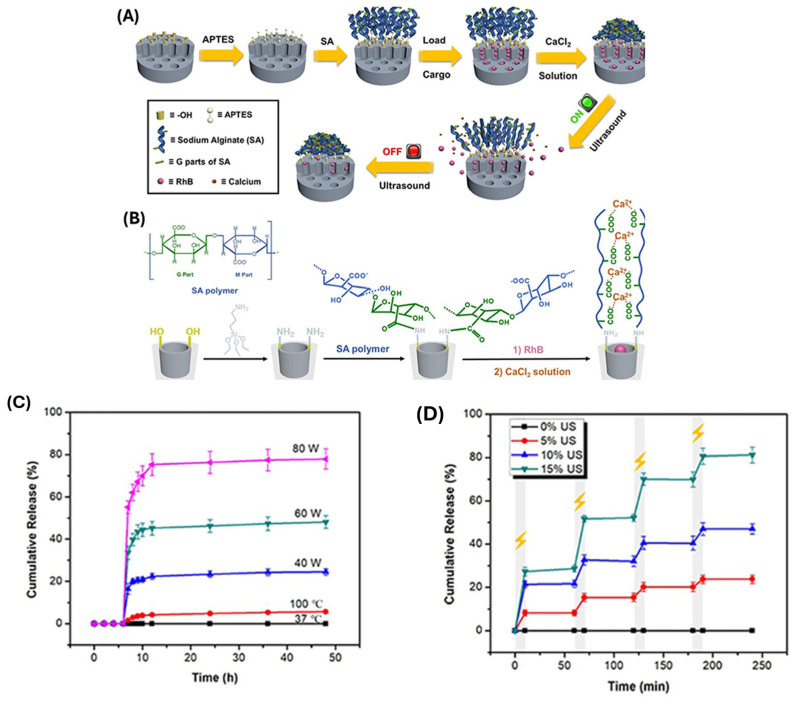
(**A**) Schematic illustration of the preparation process and ultrasonic reversible responsive behavior of the dynamic cross-linked network on hybrid MSN nanoparticles. (**B**) Molecular structure of sodium alginate (SA) and the synthetic route of calcium ion cross-linked MSN-SA. (**C**) Nature release profiles (release at 37 °C) after different treatments for 5 min (40, 60, and 80 W of HIFU and heating at 100 °C). (**D**) On-off releasing profiles at different outputs of low intensity ultrasound (0, 5, 10, and 15% of power, 10 min irradiation). ref. [99].

Li et al. [99] created a SA-MSNs with carboxyl-calcium coordination bonds in the modified layer to efficiently keep cargo inside the mesopores. The coordination bonds would be destroyed when stimulated with low intensity ultrasound (20 kHz) or high intensity focused ultrasound (HIFU, 1.1 MHz), resulting in a rapid and significant cargo release, and then reformed when ultrasound was turned off, resulting in an instant cargo release stop. The ultrasonic stimuli provided excellent real-time control of rhodamine B (RhB) release performance (Figure 7). The in vitro cytotoxicity experiment against HeLa cells was carried out with a standard MTT assay, and MSN-SA and MSN-SA@CaCl_2_ nanoparticles did not show high cytotoxicity even up to a concentration of 60 μg/mL, indicating that these two nanoparticles both had good biocompatibilities. In addition, ultrasound can cause mechanical phenomena (such as cavitation and acoustic streaming) that improve MSN cellular uptake and tissue penetration. Recent research has investigated polymer coatings made from PNIPAM, PLGA, and other smart materials that respond to ultrasound-triggered thermal or mechanical stimuli [100]. These systems have shown promise in delivering chemotherapeutics, genes, and proteins in a regulated way, especially in cancer therapy and localized disease treatment. The combination of ultrasound-responsive polymers and MSNs provides a versatile and controllable strategy for targeted drug delivery, with further advancements in polymer engineering and ultrasound technologies expected to improve the specificity, efficiency, and clinical applicability of these nanocarriers.

(vii) Dual and multi-stimuli responsive systems

Dual and multi-stimuli-responsive drug delivery systems represent an advanced class of smart nanocarriers that offer enhanced control, precision, and selectivity in drug release by responding to two or more specific physiological or externally applied stimuli [101]. In the context of polymer-modified MSNs, these systems combine the intrinsic advantages of MSNs, such as high surface area, tunable pore size, and excellent biocompatibility, with the versatility of responsive polymers to achieve sophisticated drug delivery profiles tailored to complex biological environments [102].

Dual stimuli-responsive systems typically integrate internal triggers, such as pH and redox potential, or combine an internal with an external trigger, such as temperature or light [103]. For instance, a pH/redox-responsive MSN can remain stable in the bloodstream (neutral pH and low glutathione concentration) but release drugs selectively in the acidic and reductive tumor microenvironment [104]. This synergistic response ensures minimal premature drug leakage and maximizes therapeutic action at the diseased site.

**Figure 8:**
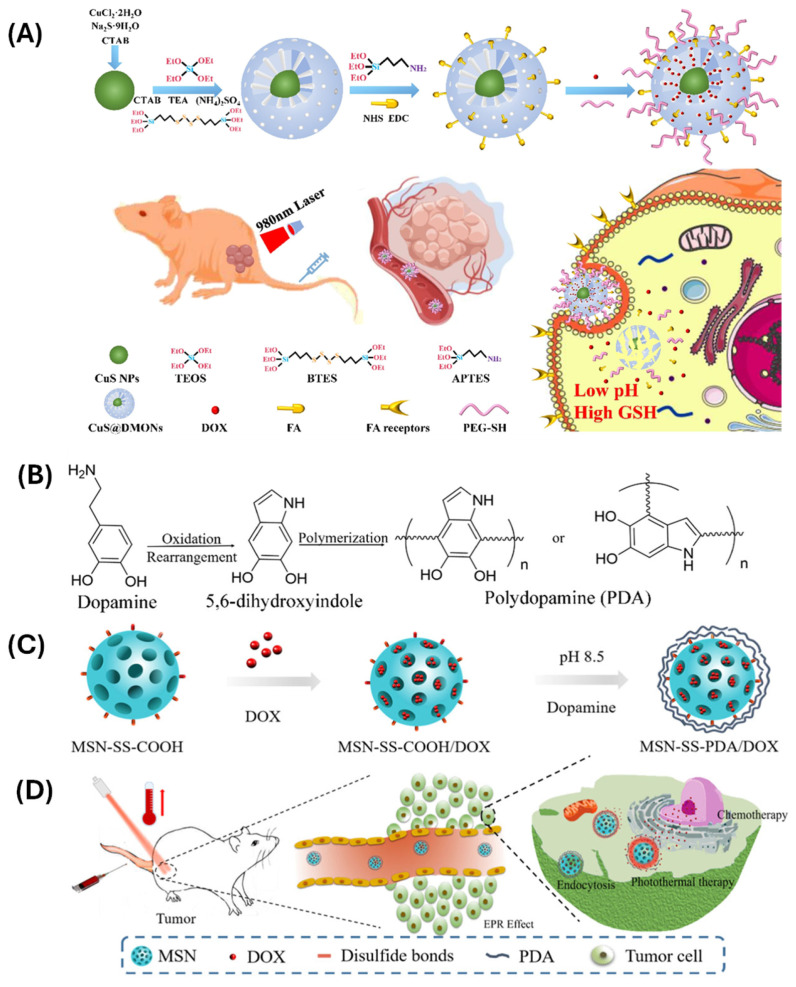
(**A**) Schematic diagram of the synergistic chemo-photothermal therapy treatment with CuS@DMONs-FA-DOX-PEG nanodrugs. Reproduced with permission from Elsevier, 2024, ref. [105]. (**B**) Synthesis of PDA film through oxidative polymerization and Michael addition reaction. (**C**) The synthesis route of MSN-SS-PDA/DOX. (**D**) The combined chemo-photothermal therapy on tumor. Reproduced with permission from Elsevier, 2019, ref. [107].

Polymers used in such systems include combinations of PAA for pH sensitivity, disulfide-linked polyethylene glycol (PEG-SS) for redox sensitivity, and PNIPAM for temperature responsiveness. These polymers can be layered or copolymerized to create integrated, multi-functional nanocarriers. Liang et al. [105] created a biodegradable NDS with a tetrasulfide bond structure, copper sulfide core, and folic acid-modified surface (CuS@DMONs-FA-DOX-PEG) to target a tumor location, dissolve, and release the drug. The CuS@DMONs-FA-DOX-PEG nanoparticles were tested for degradation ability, photothermal conversion ability, hemocompatibility, and anti-tumor activities, both in vitro and in vivo. Their findings showed that nanoparticles encapsulated in copper sulfide had high photothermal activity and an ideal photothermal conversion rate, and the drug was correctly transported and released into the target tumor cells (Figure 8A).

Regarding the cytotoxicity of CuS@DMONs-FA-PEG nanoparticles to L929, 4 T1, and A549 cells, without drug-loading, the cell survival rates were 80.88 ± 1.84, 81.27 ± 1.89, and 82.27 ± 2.42% at a concentration of 500 μg/mL, indicating that CuS@DMONs-FA-PEG empty carriers and irradiation with NIR light sources were not significantly toxic to these three cells. More complex systems also incorporate enzyme-responsive motifs or light-sensitive groups for further regulation.

Recent innovations include triple or multi-stimuli responsive MSNs, which can sequentially or simultaneously respond to a combination of pH, redox, temperature, enzymatic activity, and light [106]. Such multi-modal systems offer a high degree of spatiotemporal precision and are particularly advantageous in heterogeneous tumor environments where a single stimulus may not suffice to trigger effective drug release.

Lei and colleagues [107] developed a biocompatible multiple-sensitivity drug delivery system (DDS) by forming disulfide bonds between a polydopamine (PDA) layer and a doxorubicin (DOX)-loaded mesoporous silicon nanoparticle (MSN). PDA served as both a photothermal treatment (PTT) agent and a gatekeeper to restrict drug release. The DDS showed excellent monodispersity, redox/pH/NIR-dependent release characteristics, remarkable photothermal conversion efficiency (η = 40.21%), and outstanding tumor cell synergistic killing efficiency of chemotherapy and photothermal therapy (combination index CI = 0.175) (Figure 8B–D). The dual and multi stimuli-responsive polymer-modified MSNs provide a powerful platform for precision medicine. Their ability to integrate multiple environmental cues into a unified drug delivery system enhances therapeutic efficacy, reduces systemic toxicity, and opens up new avenues for personalized and targeted treatment strategies.

In recent studies, polymer-functionalized MSNs have emerged as highly promising carriers for the delivery of biologicals, such as vaccines and protein-based drugs. Functionalization with stimuli-responsive polymers (e.g., pH-, temperature-, or enzyme-sensitive) allows MSNs to protect sensitive biological molecules from degradation during circulation and to release their payload in response to specific physiological cues. For protein drugs and vaccines, this targeted and controlled release is critical to preserving their structural integrity and biological activity [108]. Additionally, surface modifications with polymers such as polyethylene glycol (PEG) can prolong circulation time by reducing opsonization and immune clearance. In vaccine delivery, MSNs can be engineered to co-deliver antigens and adjuvants, enhancing immunogenicity and enabling controlled immune activation [109]. Continued research into polymer-MSN systems is expanding their potential in clinical applications, especially for precision medicine and advanced immunotherapies [110].

The integration of multiple stimuli in drug delivery systems offers synergistic effects that significantly enhance therapeutic precision and efficacy. In MSNs, combining internal (e.g., pH, redox) and external (e.g., light, temperature) stimuli enables more robust control over drug release. For example, in a tumor microenvironment, acidic pH can partially trigger polymer swelling, while elevated glutathione levels simultaneously cleave disulfide bonds, resulting in a more efficient and localized drug release [111]. When combined with external stimuli, such as near-infrared (NIR) light or heat, the system can further amplify release kinetics on demand. This multi-trigger approach minimizes premature leakage, enhances spatial and temporal precision, and improves cellular uptake and endosomal escape.

Multi-stimuli-responsive drug delivery systems offer significant advantages over mono-stimuli systems by providing enhanced specificity, precision, and control over therapeutic release. While single-stimulus systems rely on one environmental or external trigger, they may face challenges such as variability in stimulus strength, premature activation, or insufficient specificity in complex biological environments [112]. In contrast, multi-stimuli systems require the simultaneous or sequential presence of two or more distinct triggers, such as acidic pH and elevated redox potential or heat and light, to initiate drug release. This dual-trigger requirement minimizes off-target effects and ensures that release occurs predominantly at the diseased site, such as a tumor [113].

## 6. Biological Considerations and Applications

### 6.1. Cellular Uptake and Intracellular Trafficking

Cellular uptake and intracellular trafficking are important factors in determining the therapeutic effectiveness of polymer-modified MSNs for stimuli-responsive drug delivery. The ability of these nanocarriers to reach target cells, penetrate them efficiently, and release their payload at the proper intracellular location is critical for accurate and successful therapy. MSNs are often ingested by cells through endocytosis, with common mechanisms including clathrin-mediated endocytosis, caveolae-mediated endocytosis, and micropinocytosis [114]. Several parameters can impact the specific pathway of absorption on the MSN surface, including size, shape, surface charge, and the type of polymer coating used.

**Figure 9:**
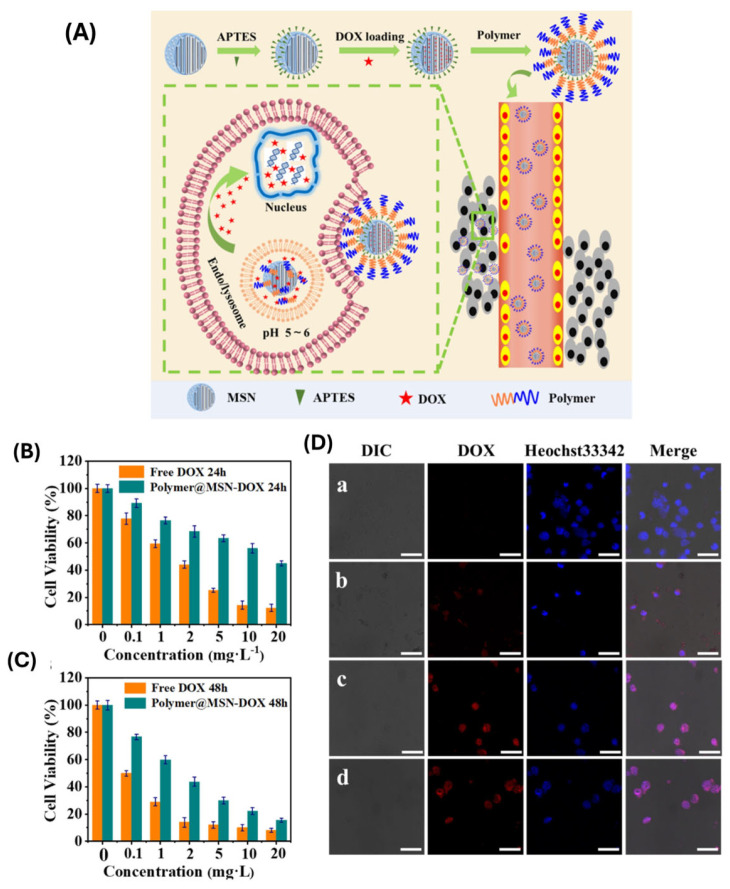
(**A**) Schematic illustration of the preparation of Polymer@MSN-DOX and pH-triggered release of DOX. (**B**) The in vitro cytotoxicity of free DOX and Polymer@MSN-DOX against HepG2 cells for 24 h (**A**) and 48 h (**B**), and CLSM images of HepG2 cells (**C**) incubated without Polymer@MSN-DOX. (**D**) (**a**) or with Polymer@MSN-DOX for 1 h (**b**) and 4 h (**c**), and with free DOX for 4 h (**d**). Reproduced with permission from Elsevier, 2019, ref. [115].

Polymer functionalization enhances colloidal stability and biocompatibility but also significantly impacts cellular interactions. For example, surface functionalization with polyethylene glycol (PEG) reduces opsonization and prolongs circulation time, while targeting ligands such as hyaluronic acid, folic acid, or peptides can be conjugated to facilitate receptor-mediated endocytosis into specific cell types, such as cancer cells overexpressing CD44 or folate receptors. Peng and coworkers [115] synthesized Schiff-base copolymer-coated mesoporous silica nanoparticles (Polymer@MSN) using ARGET ATRP and the sol-gel technique. Imine bonds in the coated polymer worked as a pH-cleavable linker between copolymer gatekeepers and MSN, promoting DOX’s controlled-release functionality. DOX released 45% more within 72 h at pH 5.0 than at pH 7.4, indicating pH-responsive drug release. Confocal microscopy investigations and in vitro cytotoxicity results demonstrated that Polymer@MSN-DOX could easily enter HepG2 cells, release DOX, and exhibit strong cytotoxicity (Figure 9).

Once internalized, MSNs are typically trafficked through the endosomal-lysosomal pathway, which presents a mildly acidic and enzyme-rich environment. This internal milieu can cause stimuli-responsive polymers to undergo structural changes or disintegration, resulting in regulated drug release within endosomes or lysosomes. For example, pH-sensitive polymers, such as poly(acrylic acid) or chitosan, can expand or disintegrate in acidic environments, whereas redox-sensitive polymers can respond to high intracellular glutathione levels by releasing the medication straight into the cytoplasm. Moreover, recent strategies aim to enable endosomal escape to prevent lysosomal degradation of sensitive cargo, such as proteins or nucleic acids. Polymers with proton-sponge effects or fusogenic properties have been integrated into MSN systems to disrupt endosomal membranes and facilitate cytoplasmic delivery [116].

### 6.2. Targeting Strategies

Targeting strategies are a critical component in enhancing the specificity and therapeutic efficacy of polymer-modified MSNs for drug delivery under stimulus-triggered conditions. These strategies aim to guide nanocarriers to diseased tissues, such as tumors or inflamed sites, while minimizing their accumulation in healthy organs, thereby reducing systemic toxicity and improving treatment outcomes. Broadly, targeting can be classified into passive and active approaches.

Passive targeting takes advantage of the increased permeability and retention (EPR) effect, in which nanoparticles aggregate preferentially in tumor tissues due to leaky vasculature and inadequate lymphatic outflow [117]. Polymer modifications, such as PEG coating, can extend the circulation period of MSNs by inhibiting fast clearance by the mononuclear phagocyte system (MPS), increasing the possibility of passive accumulation at the target location. Active targeting, on the other hand, involves functionalizing the MSN surface with specific ligands that identify and bind to overexpressed receptors on target cells. Common ligands include folic acid for targeting folate receptors, hyaluronic acid for CD44, arginine-glycine-aspartic acid (RGD) peptides for integrin receptors, and antibodies or aptamers directed against tumor-specific antigens [118].

**Figure 10:**
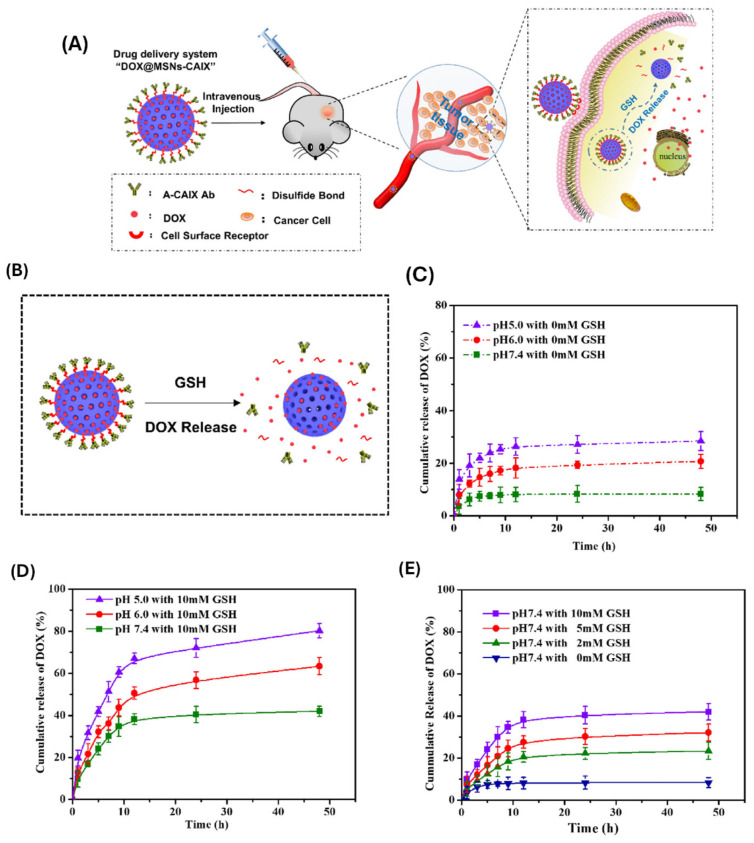
(**A**) Illustration of A-CAIX Ab targeted mesoporous silica nanoparticles as a redox-responsive drug delivery system. (**B**) GSH-triggered DOX release from DOX@MSNs-CAIX. DOX release from DOX@MSNs-CAIX in PBS with (**C**) 0 mM GSH. (**D**) 10 mM GSH at different pH values (5.0, 6.0, 7.4). (**E**) DOX release from DOX@MSNs-CAIX in PBS at pH 7.4 with different concentrations of GSH (0 mM, 2 mM, 5 mM, 10 mM). ref. [119].

These ligands facilitate receptor-mediated endocytosis, enhancing the incorporation of MSNs into target cells and improving intracellular drug delivery. Chen and colleagues [119] created a novel antibody-targeted and redox-responsive drug delivery system called “MSNs-CAIX” by attaching anti-carbonic anhydrase IX antibody (A-CAIX Ab) to the surface of MSNs via disulfide linkages. In vitro, CAIX capped doxorubicin hydrochloric (DOX)-loaded nanoparticles (DOX@MSNs-CAIX) demonstrated excellent redox-responsive release in the presence of GSH due to disulfide bond breaking. In vivo tumor targeting experiments convincingly indicated that DOX@MSNs-CAIX accumulates in tumors and induces greater tumor cell death in 4T1 tumor-bearing mice (Figure 10).

**Figure 11:**
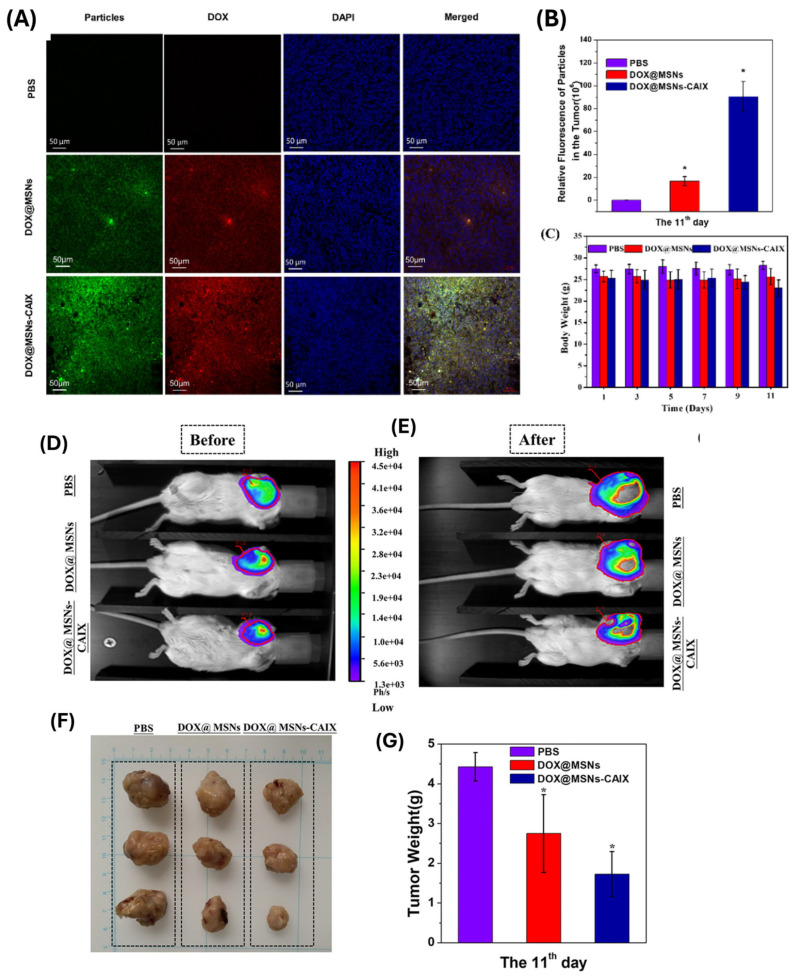
(**A**) 10 mM GSH at different pH values (5.0, 6.0, 7.4). (**B**) DOX release from DOX@MSNs-CAIX in PBS at pH 7.4 with different concentrations of GSH (0 mM, 2 mM, 5 mM, and 10 mM). (* *p* < 0.05 as compared with PBS group). (**C**) Particles and DOX distribution in tumors after being treated with the above samples for 11 days (green: particles labeled by FITC; red: DOX; blue: cell nucleus staining by DAPI). (**D**) Particles relative fluorescence intensity in the tumor. (**E**) The bioluminescence intensity imaging of the tumors before (the 0th day) and after (the 11th day) intervention with the above samples. (**F**) The final average tumor weight. (**G**) The variation curves of average tumor volume. (* *p* < 0.05 as compared with PBS group). ref. [119].

In addition, some advanced targeting systems incorporate stimuli-responsive ligands that become active only under certain conditions, such as acidic pH or specific enzymes, thus offering dual control through both targeting and release [120]. Multivalent ligand presentation has also been explored to increase binding affinity and selectivity toward diseased cells. Furthermore, dual-targeting strategies that combine passive and active targeting mechanisms are gaining attention for their synergistic benefits [121]. Long-circulating PEGylated MSNs functionalized with tumor-targeting peptides, for example, can accumulate in tumors via the EPR effect before binding to tumor cells selectively via receptor-ligand interactions (Figure 11).

### 6.3. In Vitro and In Vivo Performance

In vitro and in vivo performance studies are required to validate the therapeutic potential of polymer-modified MSNs in stimuli-responsive drug delivery. These assessments help determine the safety, efficacy, and site-specific drug release behavior of the nanocarriers under biologically relevant conditions and are crucial steps toward clinical translation.

In vitro studies are typically the first phase of performance evaluation, focusing on cellular uptake, cytotoxicity, drug release kinetics, and stimuli-responsiveness in controlled environments. Polymer-modified MSNs often exhibit enhanced cellular internalization, particularly when functionalized with targeting ligands, such as folic acid, hyaluronic acid, or peptides [122]. These systems demonstrate minimal cytotoxicity in the absence of stimuli, ensuring stability and biocompatibility during circulation. Drug release is greatly enhanced in response to certain stimuli, such as acidic pH, high glutathione content, enzyme activity, or external triggers like light or heat, validating these systems’ on-demand characteristics. Cell viability assays (e.g., MTT, live/dead), fluorescence microscopy, and flow cytometry are commonly used to evaluate internalization efficiency, targeted delivery, and therapeutic effects in cancer or disease-relevant cell lines [123].

In vivo performance, assessed using animal models, provides a more comprehensive understanding of pharmacokinetics, biodistribution, therapeutic efficacy, and potential toxicity. Polymer-functionalized MSNs demonstrate prolonged circulation times due to surface modifications such as PEGylation, and enhanced accumulation at tumor or disease sites via passive and active targeting mechanisms [124]. In tumor-bearing mice models, stimuli-responsive MSNs exhibit better tumor regression and less systemic toxicity than free drugs or non-targeted carriers. Imaging methods such as fluorescence imaging, MRI, and PET have been used to track biodistribution and real-time drug release. Additionally, histopathological analyses confirm minimal damage to healthy tissues, highlighting the safety profile of these systems.

**Figure 12:**
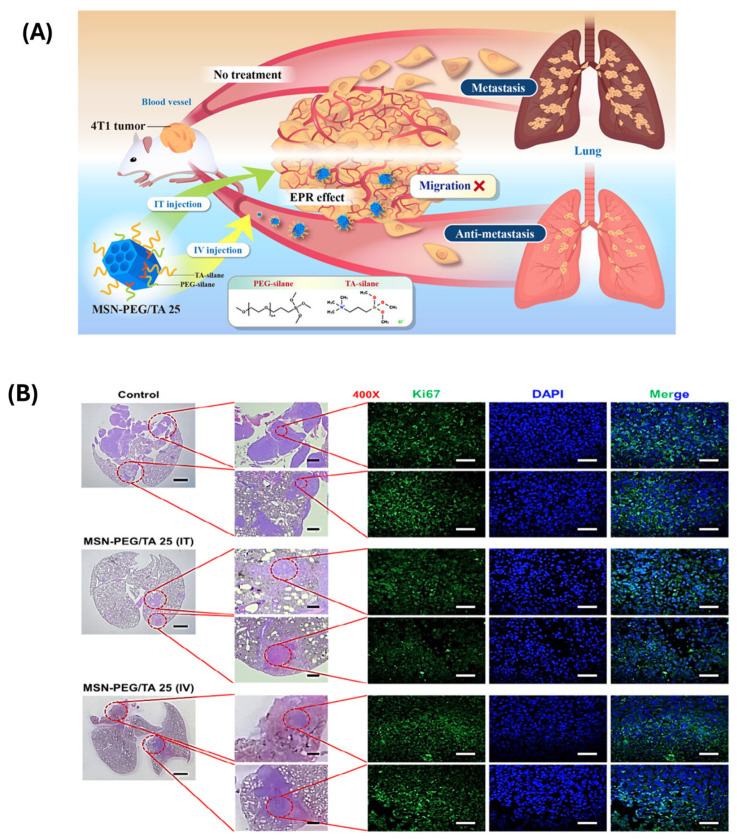
(**A**) Scheme represents the preparation of MSN-PEG/TA 25, with proper surface modifications and its utilization. (**B**) H&E-stained sections of the metastatic lung were photographed (scale bar: 500 μm). Fluorescence images of sections were stained with Ki-67 (shown in green) for metastatic nodules in the lung and DAPI (shown in blue) for nuclei (scale bar: 50 μm). 2024, ref. [125].

**Figure 13:**
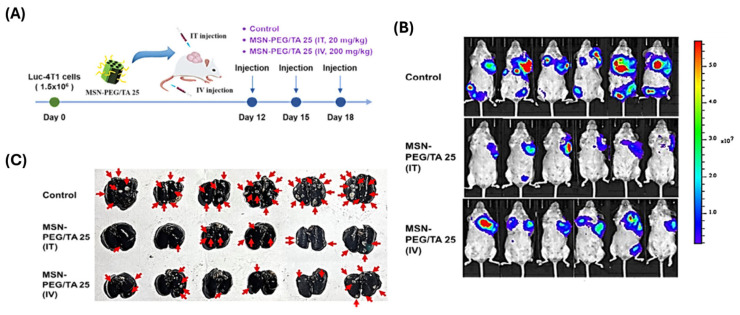
(**A**) Experimental scheme. Mice were implanted with 1.5 × 10^6^ Luc-4T1 cells and then intratumorally (20 mg/kg) and intravenously (200 mg/kg) injected with MSN-PEG/TA 25 three times. At the end point (day 35), (**B**) IVIS images of a tumor and (**C**) an India ink-stained lung (front) were taken. Red arrows point to white metastatic nodules in the lung (*n* = 6). ref. [125].

For example, Lee et al. [125] investigated the ability of mesoporous silica nanoparticles (MSN-PEG/TA 25) to decrease cancer metastasis. MSN-PEG/TA 25 coupled with liposomal-encapsulated doxorubicin (Lipo-Dox) dramatically improved mice survival rates, exceeding Lipo-Dox alone. They demonstrated that enhanced survival was due to MSN-PEG/TA 25’s antimetastatic properties, and that Dox-loaded MSN-PEG/TA 25 decreased primary tumors while maintaining the antimetastatic impact, hence improving treatment results and overall survival (Figure 12 and Figure 13).

Importantly, multi-stimulus responsive MSNs have shown better therapeutic effects in vivo due to their precise control over drug release in diverse physiological conditions. Dual pH/redox- or pH/enzyme-sensitive systems exhibit a higher level of selectivity and therapeutic efficacy in solid tumor models [126]. Furthermore, external triggers like NIR light or localized hyperthermia have been successfully used in conjunction with MSNs to initiate site-specific drug release with spatial precision.

PM-MSNs exhibit significant translation potential in clinical trials due to their tunable surface chemistry, biocompatibility, and multifunctionality. By integrating responsive polymers, these nanoparticles can achieve controlled drug release, enhanced circulation time, and improved targeting of diseased tissues. Their porous silica cores offer high drug-loading capacity, while polymer coatings provide stealth properties and responsiveness to physiological stimuli such as pH, temperature, or enzymes. Several preclinical studies have demonstrated promising results in cancer therapy, imaging, and regenerative medicine. As safety profiles and long-term outcomes continue to be assessed, PM-MSNs stand out as a versatile and promising platform for next-generation nanomedicines with strong potential for successful clinical translation.

Beyond MSNs, various other nanocarriers have been developed with clinically translatable potential. Liposomes (e.g., Doxil, a PEGylated liposomal formulation of doxorubicin) represent a successful example of passive targeting with prolonged circulation. ThermoDox^®^, a temperature-sensitive liposome, releases doxorubicin upon heating and has reached Phase III clinical trials (Table 3). Polymersomes and micelles also offer stimuli-responsive capabilities and are under preclinical or clinical evaluation. Although no MSN-based stimuli-responsive system has yet received full clinical approval, ongoing research and improved biocompatibility, targeting, and regulatory strategies suggest strong future potential. These intelligent systems aim to combine therapeutic precision with reduced toxicity for improved clinical outcomes.

## 7. Challenges and Future Perspectives

Despite the significant potential of PM-MSNs for stimuli-triggered drug administration, various difficulties must be overcome before their practical use. One of the most significant challenges is the intricacy of synthesis and functionalization. Obtaining exact control over particle size, shape, porosity, and homogeneous polymer coating is technically difficult and sometimes lacks scalability [127]. Batch-to-batch variation can significantly affect reproducibility, which is crucial for regulatory approval. Additionally, the integration of multiple stimuli-responsive functionalities, while beneficial for specificity, increases formulation complexity and may lead to unpredictable behavior in vivo.

Biocompatibility and long-term safety also remain significant concerns. Although silica is generally regarded as biocompatible, its degradation kinetics and accumulation in tissues require thorough investigation. Surface polymers, particularly synthetic ones, may also elicit immunogenic or inflammatory responses [128]. Comprehensive toxicological studies, including biodistribution, metabolism, and excretion, are essential to understand the long-term impact of these nanocarriers. Another difficulty is biological variability. Internal stimuli, such as pH, redox potential, or enzyme activity, can vary greatly between people, tumor types, and even within the same tumor. This heterogeneity can reduce the reliability of stimuli-responsive release mechanisms. External stimuli, such as light or heat, offer more control but may suffer from limitations in tissue penetration, especially for deep-seated tumors.

Future perspectives are focused on addressing these limitations through improved design strategies. Advancements in bioinspired polymers and degradable organic–inorganic hybrids are being explored to enhance biocompatibility and reduce long-term toxicity. Efforts are also being made to develop stimulus-amplifying systems, where small environmental cues can trigger a cascading release mechanism, improving responsiveness in heterogeneous tumor environments. The use of artificial intelligence (AI) and machine learning to optimize nanocarrier design based on biological feedback is a growing area of interest. Furthermore, personalized nanomedicine could allow the design of MSN systems tailored to an individual’s tumor microenvironment and physiological conditions. In parallel, regulatory agencies are gradually adapting guidelines for nanomedicines, which may soon provide a clearer pathway for clinical approval.

## 8. Conclusions

To summarize, PM-MSNs have emerged as extremely promising platforms for stimuli-triggered drug administration, with improved accuracy, controlled release, and low systemic toxicity. Mesoporous silica’s large surface area, variable porosity, and polymer modification adaptability make it an ideal basis for building improved drug delivery systems. MSNs can be modified to release therapeutic compounds in response to a variety of stimuli, including pH, redox potential, temperature, light, and enzymatic activity. The capacity to precisely release drugs in specific environments, such as tumor tissues or inflammatory locations, offers substantial advances in personalized medicine and precision treatments.

Recent advancements in polymer-functionalization strategies, such as the incorporation of targeting ligands, dual or multi-stimuli responsiveness, and hybrid organic–inorganic materials, have further improved the efficiency and specificity of MSNs. These innovations have paved the way for MSNs to exhibit enhanced bioavailability, better pharmacokinetics, and more effective in vivo performance. Furthermore, dual-targeting strategies that combine both passive and active targeting mechanisms, alongside stimulus-responsive drug release, have shown substantial promise in improving therapeutic outcomes while minimizing side effects. However, despite these tremendous advances, some problems persist, such as synthesis complexity, biocompatibility, in vivo variability, and regulatory approval. Addressing these concerns through multidisciplinary research, better material design, and rigorous toxicity testing will be critical to the successful implementation of these systems in clinical practice. Looking ahead, future research should focus on optimizing stability, targeting specificity, and real-time monitoring of drug release. The integration of artificial intelligence in the design of these systems, along with personalized drug delivery approaches, could further enhance the potential of PM-MSNs in clinical settings. In summary, while significant progress has been made, continued innovation and refinement of these systems will be key to realizing their full therapeutic potential in treating a variety of diseases, particularly cancer and other localized conditions.

## Figures and Tables

**Table 2:** The physical properties of various studies on MSNs.

Polymer Type	Hydrodynamic Size Range (nm)	Zeta Potential	Colloidal Stability	Biocompatibility	Stimuli-Responsiveness	Degradability
PEG (Polyethylene Glycol)	100–150	Neutral to slightly negative	High	Excellent	None	Non-biodegradable (but inert)
Chitosan	120–180	Positive	Moderate to High	Good	pH-responsive	Biodegradable
PNIPAM (Poly(N-isopropylacrylamide))	130–200	Slightly negative	High	Good	Thermo-responsive (~32 °C)	Limited
PAA (Polyacrylic Acid)	110–160	Strongly negative	Moderate	Fair	pH-responsive	Non-biodegradable
PLA (Polylactic Acid)	100–170	Slightly negative	Moderate	Excellent	Slow hydrolysis	Biodegradable
PCL (Polycaprolactone)	120–180	Slightly negative	Moderate	Excellent	None	Biodegradable
Dextran	100–160	Neutral	High	Excellent	Enzyme-responsive	Biodegradable

**Table 3:** List of ongoing clinical trials of MSNs.

Trial Identifier	Formulation	Application	Phase	Status
NCT01266096	^124I-cRGDY-PEG-C′ dots	PET imaging of melanoma and brain tumors	I	Active, not recruiting
NCT02106598	cRGDY-PEG-Cy5.5-C′ dots	Fluorescence imaging of head and neck melanoma	II	Recruiting
NCT03465618	^89Zr-DFO-cRGDY-PEG-Cy5-C’ dots	PET-CT imaging of malignant brain tumors	I	Active, not recruiting
NCT04167969	^64Cu-NOTA-PSMA-PEG-Cy5.5-C′ dots	PET/MRI-guided surgery for prostate cancer	I	Recruiting
NCT01270139	Silica-gold nanoparticles (NANOM-FIM)	Photothermal therapy for atherosclerosis	-	Completed
NCT00848042	AuroShells (silica-gold nanoshells)	Photothermal ablation of head and neck cancer	-	Completed
NCT04240639	AuroShells (silica-gold nanoshells)	MRI/US-guided photothermal ablation of prostate cancer	-	Active, not recruiting
